# Particle–Bubble Interactions: an Investigation of the Three-Phase Contact Line by Atomic Force Microscopy

**DOI:** 10.1021/acs.langmuir.3c01781

**Published:** 2023-09-14

**Authors:** Jan Nicklas, Lisa Ditscherlein, Urs A. Peuker

**Affiliations:** TU Bergakademie Freiberg, Institute of Mechanical Process Engineering and Mineral Processing, Agricolastraße 1, 09599 Freiberg, Germany

## Abstract

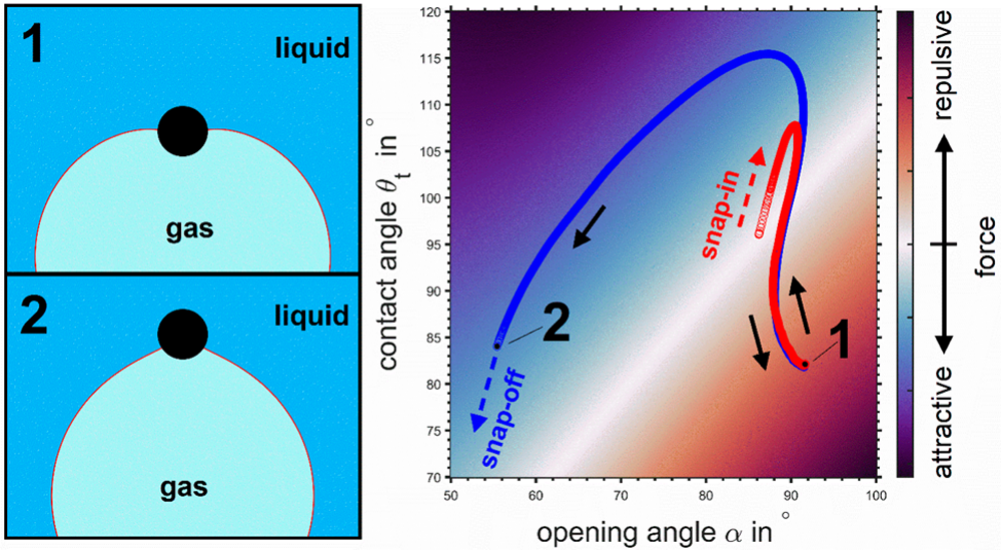

The dynamics of the three-phase contact line during particle–bubble interactions determine the stability of particle–bubble aggregates in flotation. The interaction of particles and sessile gas bubbles can be studied by colloidal probe atomic force microscopy (CP-AFM). This paper demonstrates a method to obtain the contact angle, the position of the three-phase contact line on the particle, and the bubble profile by utilizing the full information contained in AFM force–distance curves, i.e., force and CP-position information as well as the work done to move the three-phase contact line on the CP-particle. The proposed method does not require any assumption of a constant contact angle or a constant opening angle. This is achieved by the combined solution of the particle force balance and an expression for the work required to move the three-phase contact line over the colloid probe. The applicability to AFM force–distance measurements was demonstrated for the interaction of a hydrophobic SiO_2_ or a hydrophobic Al_2_O_3_ colloidal probe particle with sessile gas bubbles having radii between 45 and 80 μm.

## Introduction

In mineral flotation, the particle affinity for the gas–liquid interface after the attachment to the bubble is characterized by the contact angle θ at the particle’s wetting perimeter, the so-called three-phase contact line (TPCL). At the TPCL, the three phases of gas, particle material, and surrounding liquid meet. The relationship between the three phases at the TPCL under equilibrium conditions is described by the Young equation, eq 1, which links the surface tension γ_gl_ and the two interfacial energies of the solid–liquid γ_sl_ and the solid–gas γ_sg_ interfaces to the macroscopic contact angle θ.
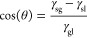
1

For a clean air–water interface, the surface tension is γ_gl_ = 72 mN/m, which in the following is denoted without indices as γ. At rest, the location of a particle inside the gas–liquid interface of a bubble is characterized by its wettability dependent equilibrium three-phase contact angle. In practice, the equilibrium three-phase contact angle of a particle is only directly accessible by imaging methods,^1^ and for small particles below 50 μm diameter it is difficult to determine this quantity reliably. Therefore, researchers often resort to the measurement of macroscopic equilibrium contact angles or macroscopic dynamic contact angles on flat substrates by either the sessile drop method^2^ or the captive bubble method.^3^ The comparability to contact angles determined by nonimaging methods such as the AFM^4,5^ or the capillary rise method^5^ is rarely given.

Ducker^6^ performed the first atomic force microscopy (AFM) experiments between sessile gas bubbles with radii *R*_b_ ≈ 200–300 μm and either hydrophilic or hydrophobic SiO_2_ colloidal-probe particles with radii *R*_CP_ = 3–5 μm. Soon after, the characterization of particle–bubble interactions by AFM before^7,8^ and after particle attachment^4,5^ to the interface gained notable attention.

It was suggested that advancing and receding contact angles during the interaction of a particle with a gas bubble can be indirectly determined from AFM data,^4,5,9−11^ without imaging of the bubble interface profile. The theories that are applied to determine advancing and receding contact angles from AFM-measurements are exclusively based on models for flat air–water interfaces and do not account for the curvature of the gas bubble during the interaction, which becomes critical when the radii of the particle and the sessile gas bubble are in a comparable size range. This limits the interpretability of particle–bubble AFM measurements and leads to inconsistencies, which fail to explain the frequently reported contact angle hysteresis^5,10^ that is observed when the direction of the CP-particles movement is reversed, i.e., the movement of the CP-particle changes from approach to retract. Due to the lack of a comprehensive theory that can describe the full interaction-cycle of particle-bubble contact and detachment consistently, researchers commonly resort to one of the following two assumptions: either a pinned contact line on the CP,^4^ which results in a constant opening angle α and the change in the measured force is accounted for by a change of the contact angle, or that the contact angle remains constant^1,12,13^ and the three-phase contact line (TPCL) can freely slide over the particle.

The presented paper demonstrates a method to obtain the contact angle, the TPCL-position and the bubble profile by utilizing the full information contained in AFM force–position curves, i.e., force and CP-position information, as well as the work done to move the TPLC over the CP-particle.

### Forces Acting on a Particle Attached to the Bubble Interface

**Figure 1:**
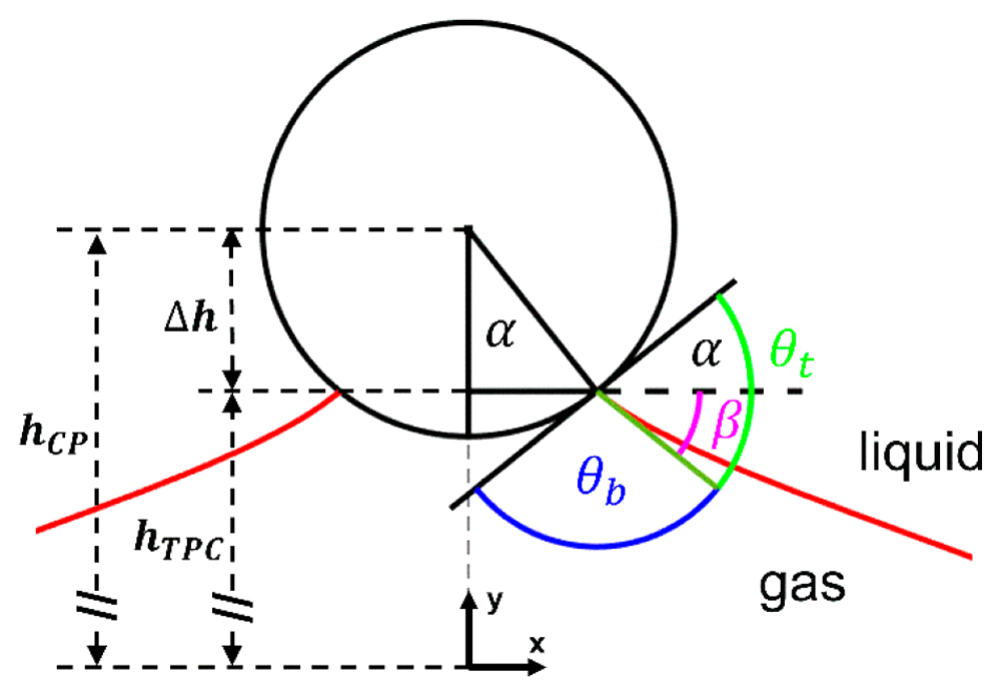
Particle located inside the gas–liquid interface of a sessile bubble. The three-phase contact of the particle–bubble aggregate is defined by the opening angle α and the inclination of the gas–liquid interface at the TPCL β. The sum of the upper phase contact angle θ_t_ and the bottom phase contact angle θ_b_ is equal to 180°. The depth of particle immersion Δ*h* is defined by the opening angle α; *h*_TPC_ is the height of the three-phase contact line (TPCL) with respect to the substrate, and *h*_CP_ is the distance between the particle center and the substrate (*y* = 0 μm).

The force balance for a particle inside the gas–liquid interface of a sessile gas bubble (Figure 1) is given by eq 2, with the vertical component of the capillary force, *F*_cap_, and the pressure force, *F*_p_ that is pushing the immersed particle out of the bubble. *F*_b_ is the buoyancy force resulting from the displaced gas volume of the immersed part of the particle, and *F*_g_ is the gravitational force. The relative importance of gravitational and capillary effects is characterized by the capillary length , which equals *l*_cap_ = 2.7 mm for water–air interfaces. The sum of the forces in eq 2 is equal to the force *F*_ext_, which is sensed by the CP-cantilever in an AFM experiment. The particles and bubbles in AFM experiments are significantly smaller than the capillary length, for this reason the buoyancy and gravitational terms in the force balance are typically neglected when AFM data is evaluated.
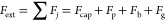
2

The vertical component of the capillary force acting on a particle inside a fluid–fluid interface is given by eq 3, in which α is the opening angle determining the position of the three-phase contact line on the particle surface via the contact radius *r*_TPC_, eq 4, and the depth of particle immersion with respect to the particle center Δ*h* that is given by eq 5. The inclination of the fluid–fluid interface at the three-phase contact line with respect to the horizontal *x*-axis is given by the angle β, which is related to the upper phase contact angle θ_t_ (by eq 6) or, alternatively, can also be expressed in terms of the bottom phase contact angle: θ_b_ = π – α – β.
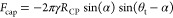
3
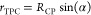
4
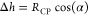
5
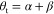
6

In summary, the capillary force given in eq 3 is defined by the surface tension γ, the particle radius *R*_CP_, and the local geometry of the TPCL, which is described by two angles, which are the opening angle α and one of the angles β, θ_t_, or θ_b_. Equation 3 is expressed in terms of the upper phase contact angle θ_t_.

Once the film rupture and initial dewetting of the particle surface have occurred and the particle is located inside the gas–liquid interface, the force balance in eq 2 under consideration of eq 3 should be valid for both the approach and retract parts of FD-AFM measurements.

### Common Theories

**Figure 2:**
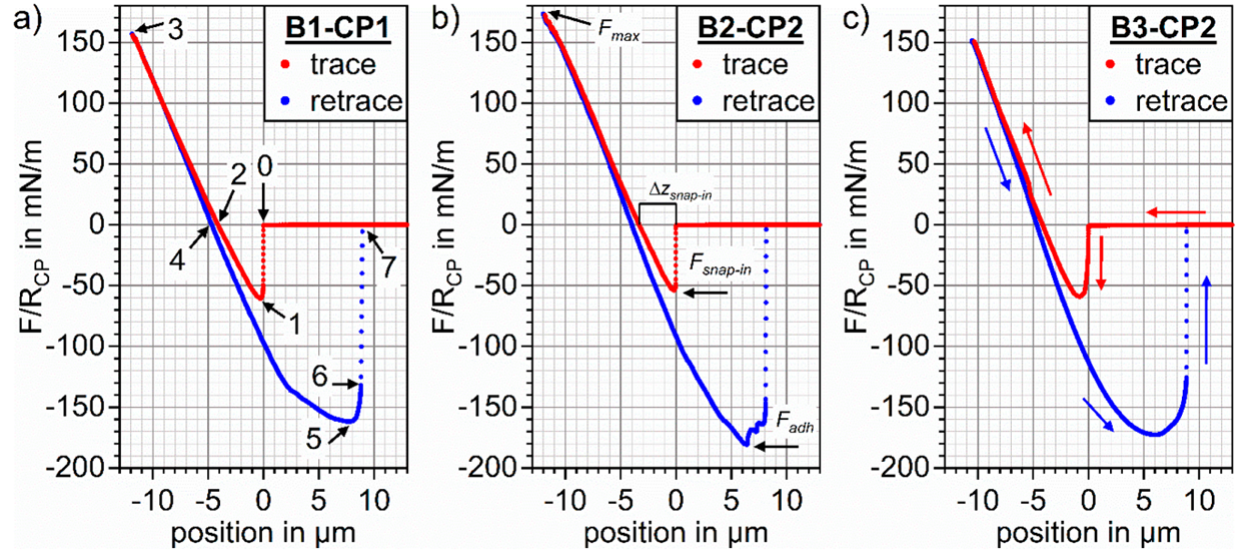
Force–position curves for the interaction of hydrophobic particles of radius *R*_CP_ with sessile gas bubbles with radius *R*_b_ at a piezo drive velocity of *v* = ±30 μm/s. The measured force *F* is scaled by *R*_CP_ and the positition is given relative to the point of first contact with the undeformed sessile bubbles: (a) Silanized SiO_2_–CP (*R*_CP_ = 5.6 μm) and *R*_b_ = 68 μm (B1-CP1); (b) silanized Al_2_O_3_–CP (*R*_CP_ = 7.2 μm) and *R*_b_ = 80 μm (B2-CP2); (c) silanized Al_2_O_3_–CP (*R*_CP_ = 7.2 μm) and *R*_b_ = 45 μm (B3-CP2). The numbers 1–6 in (a) correspond to the *X*–*Y* trajectories in Figure 5 and the calculated bubble–interface profiles that are shown in Figure 10.

The contact angle upon attachment, θ_at_, is frequently obtained by using eq 7, where *R*_CP_ is the radius of the colloidal probe particle and Δ*z*_snap-in_ is the distance between the zero crossing at the snap-in (Figure 2b) and the so-called “equilibrium position”.^4,14^ In eq 7 it is assumed that *F* = 0 nN, the gas–liquid interface is flat, and therefore β = 0 and θ_t_ = α.
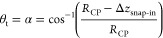
7

Equation 8 relates the maximum adhesion force, *F*_adh_, during retraction of a particle from a flat gas–liquid interface and the contact angle upon detachment under the assumption of a free movement of the contact line along the perimeter of the particle.^15^ It was first derived by Nutt^16^ and shortly after in more detail by Scheludko and Nikolov.^15^Equation 8 corresponds to the situation where α = β = θ_t_/2 and can be obtained by inserting this relationship between the angles into eq 3.
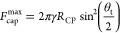
8

This widely spread theory for particles located inside fluid–fluid interfaces was applied to experiments with millimeter sized particles and its applicability to microparticles immersed in a flat gas–liquid interface was recently tested by Schellenberger,^1^ by combining AFM force spectroscopy with film height measurements via confocal microscopy for the interaction of a silica colloidal probe (*R*_CP_ = 6.9 μm) and a film of glycerol. Schellenberger observed a pinned contact line and therefore an almost constant opening angle during particle retraction until the maximum pull-off force was reached, but concluded that the experiment is likely not representative for dynamic interactions due to the time given for the glycerol meniscus to equilibrate.^1^

However, the applicability of Scheludko and Nikolov’s theory to curved interfaces of small microbubbles is still uncertain, and few approaches exist to account for the curvature of the bubble interface. Available approaches are based on free energy calculations^17−19^ and involve sophisticated numerical solution techniques, presumably giving these approaches a purely academic relevance. An approach of moderate mathematical complexity for the solution of the force balance, eq 2, under consideration of the bubble interface profile was presented by Sherman.^19^ This approach couples the force balance and a parametric representation of the Young–Laplace-Equation, eq 9. Sherman’s approach still requires the measurement of interface profiles during the interaction, as the number of unknown parameters is too high to solve the problem with the parameters supplied by only considering force position data.
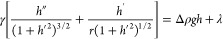
9

In the modified Young–Laplace equation, eq 9,^19^*h*(*r*) is the height of the bubble interface along the radial coordinate *r*, *h*′ = d*h*(*r*)/d*r* and *h*″ = d^2^*h*(*r*)/d*r*^2^ are the corresponding derivatives with respect to *r*. λ is the Lagrange multiplier, which has the units of pressure and is required to satisfy the constant bubble volume constraint.^19^ The modified Young–Laplace equation utilizes the parametric representation introduced by Huh and Scrieven,^19,20^ with the scaled variables x* = *r*/*l*_cap_, y* = *y*/*l*_cap_ and the local inclination of the bubble profile d*h*/d*r* = tan(ϕ).
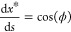
10a
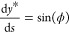
10b
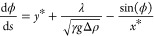
10c

The Lagrange multiplier λ is considered in the solution for the bubble profile (eqs 10a–10c), as well as in the pressure term, eq 11, of the force balance, eq 2, linking both. In eq 11, Δρ is the density difference between the two fluid phases and *h*_TPC_ is the height of the TPCL with respect to the substrate (Figure 1).
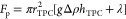
11

Sherman’s solution of eqs 10a–10c differs from the one proposed by Huh and Scriven only in the specified initial conditions, which Sherman considers to be at the TPCL position on the particle surface, specified by *x*_TPCL_^*^, *y*_TPCL_^*^, and ϕ_TPCL_ = π – β. This implies predetermined values for α, θ_t_ and β, according to eqs 10a–10c. Sherman obtained the angles that define the TPCL and λ by fitting eqs 10a–10c and eq 2 to simultaneously recorded interface profiles and force–position data.^19^

To utilize the full information contained in the force–position data, the area under the force–position curves should be considered, which is a measure for the work done on the capillary system; Schulze^21^ suggested that the work required to detach a particle from a gas bubble can be obtained by integration of the force balance, eq 2, with respect to the deflection of the bubble interface from its undeformed equilibrium shape. This is equivalent to an integration over the height of the TPCL *h*_TPC_ with respect to the substrate and therefore can be written as
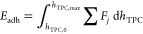
12

Integrating the sum of forces with respect to *h*_TPC_ neglects the dependence of the TPCL position on the opening angle α. This approach utilizing the work of detachment *E*_adh_ is to date the foundation for the detailed assessment of particle–bubble–aggregate stability in flotation.^22^ It also provides the idea for the available closed-form approximations for the work of particle detachment from flat interfaces.^12,23,24^ Both Pitois^25^ and Anachkov^12^ obtained expressions for *E*_adh_ by rewriting the integral in eq 12 in terms of the opening angle α, however, always under the assumption of a constant contact angle. Recent tensiometer studies^1,26^ that combine force–distance-measurements and high-speed camera recordings of the interface profile during particle–bubble interaction suggest that neither the condition of a constant opening angle, nor a constant contact angle are fulfilled during particle retraction.

## Materials and Methods

### AFM Measurements

Colloidal probe–atomic force microscopy (CP-AFM) measurements on sessile gas bubbles were carried out in contact mode using an XE-100 (Park Systems, Korea), equipped with a long-range scan head that has a 25 μm piezo z-scan range. For colloidal-probe particles (CP particles) either a hydrophobized SiO_2_ microsphere (SiO_2_-F-SC98-7, microparticles GmbH, Germany) or a hydrophobized Al_2_O_3_ particle (DAW-07, Denka, Japan) were used (Table 1). Particle hydrophobization was achieved by silanization with the silane 1*H*,1*H*,2*H*,2*H*-perfluorooctyltriethoxysilane (AB104055, C_14_H_19_F_13_O_3_Si, abcr, Germany). After cross-linking of the silane at 120 °C in a drying oven, the CP particles were washed in high purity ethanol ≥99.9% (LiChrosolv Merck, Germany) to remove any free silane molecules that could act as contaminants. The CP particles were glued onto AIOTL-C cantilevers (Budged Sensors, Bulgaria) with water-insoluble epoxy resin (UHU Endfest 300, UHU, Germany). The theoretical cantilever spring constants were calculated from each cantilever resonance frequency under the assumption of a rectangular beam shape, using the method described by Butt,^27^ and were in the range *k*_*c*_ = 6.8–6.9 N/m (Table 1). Calibration of the CP-cantilevers was done through the repeated measurement of force–position curves for the interaction of the CP with a naturally oxidized Si-wafer immersed in ultrapure water (Milli-Q RefA+, Merck, Germany). The calibration measurements were repeated, and the AB-sensitivity was adjusted, until the deviation between the slope of the force–position curve and the theoretical spring constant was below 1%.

**Table 1:** Experimental Parameters for the CP-AFM Experiments are Shown in Figure 2 and the Resulting Key Characteristics That Were Extracted from the Force–Position Data^a^

label	*R*_b_ in μm	Δ*P*_L_ in Pa	CP No.	*k*_c_ in N/m	*R*_CP_ in μm	CP material	Δ*z*_snap-in_ in μm	*F*_adh_/*R*_CP_ in mN/m			*F*_snap-in_/*R*_CP_ in mN/m			*E*_adh_/*R*_CP_^2^ in mN/m		
								min	max	median	min	max	median	min	max	median
B1-CP1	68	2100.6	1	6.8	5.6	SiO_2_-sil	4.1	160.8	166.9	161.8	58.6	66.8	60.6	260.4	274.8	268.4
B2-CP2	80	1785.5	2	6.9	7.2	Al_2_O_3_-sil	3.4	180.5	183.6	181.6	49.9	60.9	53.9	183.7	207.3	193.5
B3-CP2	45	3174.2	2	6.9	7.2	Al_2_O_3_-sil	4.2	160.0	184.6	172.7	37.6	67.6	59.1	181.3	262.4	224.9

a The different experiments are labelled according to the bubble (B) and the CP-particle it interacts with (CP). *R*_b_ is the bubble radius, Δ*P*_L_ is the corresponding Laplace pressure, *R*_CP_ is the radius of the CP-particle, and the spring constant is *k*_c_. *F*_adh_ is the maximum pull of force, *F*_snap-in_ is the force measured at the snap-in, and *E*_adh_ is the work of adhesion taken from the integral under the retrace-curves of the individual measurements, all scaled by the radius of the CP-particle *R*_CP_.

The electrolyte solution for the measurements was prepared 1 day in advance with ultrapure water and a mixture of 1 mmol/L sodium nitrate NaNO_3_ (Roth, Germany) and 1 mmol/L sodium chloride NaCl (Roth, Germany). Sessile gas bubbles with 90 to 160 μm diameter were generated by an ethanol solvent exchange in an electrolyte solution. For this, 1 μL of high purity ethanol ≥99.9% (LiChrosolv, Merck, Germany) was placed on the substrate (silanized Si-wafer), which was subsequently immersed into the solution. The immersion is followed by gas supersaturation and the subsequent growth of sessile microbubbles on the substrate. The surface tension of the measurement solution under consideration of the ethanol contamination (0.033 vol %) was determined by the Wilhelmi plate method (Tensiometer K12, Krüss, Germany) and is γ = 71.42 mN/m. This value for γ is used in all calculations.

For the AFM measurements, the CP cantilever was concentrically aligned with the sessile gas bubbles using the top-view light microscope of the AFM, which also provided the diameter of the sessile bubbles. The initial distance between the lower end of the CP and the undisturbed bubble interface was adjusted to about 15 μm using the integrated stepper motor. A minimum of 150 consecutive force–position curves were measured per bubble, utilizing the long-range piezo that was displaced at a velocity of *v* = ±30 μm/s. The force–position curves were aligned at the snap-in to counteract the displacement of the bubble TPCL with the substrate. To facilitate this step, the experimental raw data was mapped onto an equidistant position grid, i.e., the data points of the experimental force–position curves are interpolated and evaluated at identical positions. The median force is then calculated at each point along the equidistant grid from the set of shifted force–position curves, giving the median force–position curve for each experimental run (Figure 2).

### Mathematical Model

Starting point of the proposed method for the calculation of contact angle, TPCL position, and the bubble profile from AFM force–position data are the ideas of Sherman^19^ and Schulze.^21^ For a given set of experimental AFM force position measurements, we aim to simultaneously solve the force balance, eq 2, and an expression for the work done on the capillary system equivalent to eq 12, while maintaining the link to the current bubble interface profile described by eqs 10a–10c. The connection is achieved by utilizing parameter λ, which is required to meet the constant volume constraint for the sessile bubble. The sizes of the particles (*R*_CP_ < 10 μm) and sessile bubbles (*R*_b_ < 100 μm) that are investigated in this paper are much smaller than the capillary length, which permits the simplification of the force balance, eq 2, by neglecting *F*_b_ and *F*_g_. Therefore, the force acting on the CP particle *F*_ext_ is given by eq 13, and eq 14 is obtained in analogy to Shermans force balance^19^ without considering the buoyancy term accounting for the liquid volume that is displaced by the CP particle.
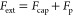
13
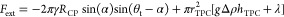
14

By including the geometrical relationship, *h*_TPC_ = *h*_CP_ – *R*_CP_ cos(α) (Figure 1) between the height of the CP center, *h*_CP_, with respect to the substrate and the height of the TPCL, *h*_TPC_, we obtain eq 15 as a function of α, θ_t_, λ and *h*_CP_.
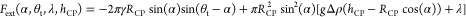
15

In previous studies utilizing the work during particle detachment from a gas–liquid interface according to eq 12, the work is obtained by integration of the force, eq 2 or eq 3, starting from the particles “equilibrium position”, where the measured force is equal to zero, up to a critical displacement of the particle from its initial position.^12,21,25^ Considering the CP-displacement or the CP-position, with respect to the undeformed bubble interface, is equivalent to considering a global coordinate system with its origin at the height of the substrate, on which the sessile bubble is located. We pursue a slightly different approach and instead define a local coordinate system located at the CP center as the point of reference and therefore consider the work required to move the TPCL up and down on the CP particle.

The height of the TPCL on the CP with respect to its center is given by eq 5 or eq 16. A positive sign of Δ*h* therefore means that the TPCL is located on the lower half of the CP-Particle (α < 90°), whereas Δ*h* < 0 indicates that the TPCL is located above the particle center (α > 90°). By rearranging eq 5, we can express the opening angle α in terms of Δ*h* (Figure 1) by eq 17.
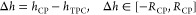
16
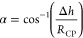
17

This relationship is inserted into the force balance eq 15 to obtain eq 18, which now is a function of Δ*h*, θ_t_, *h*_CP_, and λ. The parameter λ has the units of pressure and typically its value is close to the Laplace pressure  of the gas bubble under consideration. For the undeformed bubble without an attached particle λ ≈ Δ*P*_L_, and for the bubble in contact with the particle λ ≥ Δ*P*_L_. However, the increased value of λ does not exceed the equilibrium Laplace pressure by more than 12.5% when solving eqs 10a–10c under consideration of the constant volume constraint for bubble interfaces that are stretched up to 10 μm from their undeformed position. It follows that the interaction between microbubbles of a size below the capillary length *R*_b_ < *l*_cap_ and micrometer-sized particles, is governed by both the capillary force and the excess pressure due to the bubble curvature, which is accounted for by λ, because *g*Δρ(*h*_CP_ – Δ*h*) ≪ λ.
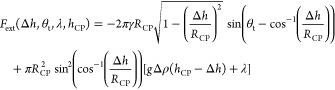
18This permits us to consider only the capillary force *F*_cap_ and the force *F*_λ_ due to the excess pressure component λ in the subsequent analysis:
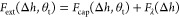
19
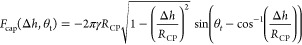
20
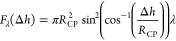
21

### Large Gas Bubble–Flat Interface

For the sake of clarity, we first consider the scenario of a big gas bubble with an approximately flat interface, for which the additional pressure due to the curvature of the bubble is negligible (λ → 0) and only the capillary force component has to be considered, given that the particle radius is significantly smaller than the radius of the bubble. Hence, the force acting on the CP particle, not taking into account the influence of the cantilever or bubble curvature, is given by
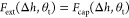
22

To obtain the expression for the work required to move the TPCL up and down the CP-particle, eq 23a, the force balance eq 22 is integrated with respect to the upper phase contact angle θ_t_ and the contact line displacement from the particle center Δ*h*. Starting point for the integration is the moment just before the snap-in, where no work has yet been done and no force is acting on the CP. Of course, this is a simplification of the real situation of particle attachment, where DLVO-forces, arguably hydrophobic forces, and hydrodynamic forces are present between CP and the gas bubble. This phase of the particle bubble interaction is fairly well understood and described by the Stokes–Reynolds–Young–Laplace model.^7^ However, the mentioned forces before the snap-in are only relevant below about 10–100 nm of separation distance, whereas the relevant length scale for the capillary interaction of μm-sized particles with a microbubble is expected to be ≥0.1 μm. The start of the integration at Δ*h* = *R*_CP_ and θ_t_ = 0 is the only reference point that does not require any elaborate assumptions about the values of θ_t_ and α. The macroscopic description of particle wetting cannot take into account asymmetries, microscopic contact angles, and dimpling or wimpling that may occur during the initial contact. Therefore, for a vanishing distance between CP and the interface, the starting point of macroscopic particle wetting is given by α = 0 and with the bubble interface being undisturbed (β = 0) at θ_t_ = 0. The condition θ_t_ = 0 is also obtained as the limiting case of a vanishing particle size *R*_CP_ → 0, and therefore, *x*_TPCL_^*^ → 0 in Sherman’s model,^19^ for which eqs 10a–10c recover the undeformed bubble profile.
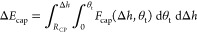
23a
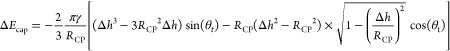
23b

### Small Gas Bubble-Curved Interface

For small gas bubbles, the excess pressure λ caused by the curvature of the interface cannot be neglected; therefore, an expression for the work similar to eq 23b is required that contains the contributions of capillary force and pressure force:
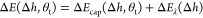
24

Δ*E*_cap_(Δ*h*, θ_t_) is already known from the situation of an interface with negligible curvature (eq 23b). The parameter λ has to be considered a macroscopic quantity, and therefore, the effect of the local curvature on its value is expected to be small; thus, we approximate λ ≈ const and assume that Δ*E*_λ_ is independent of the upper phase contact angle θ_*t*_. The integral of *F*_λ_(Δ*h*), eq 25a, leads to the expression Δ*E*_λ_ for the work required to overcome the bubble excess pressure eq 25b. The integration constant in eq 25b is obtained by imposing the condition Δ*E*_λ_(Δ*h* = *R*_CP_) = 0, meaning that at the outermost possible contact point of the CP with the bubble, the particle is not yet dewetted. The resulting expression is easily interpreted as the work required to displace the gas volume inside the bubble, as eq 25b can be rewritten to be a function of the immersed particle volume  =  that displaces the gas inside the bubble during the interaction, giving Δ*E*_λ_(*V*_cap_) = −λ*V*_cap_.
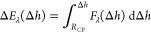
25a
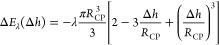
25b

### Calculation of Contact Angles from Force–Position Data

The force balance, eq 19, and the work required to move the TPCL along the particle, eq 24, form an equation system that can be solved numerically to obtain θ_t_ and Δ*h* for a given pair of *F*_ext_ and Δ*E* values, which can be extracted from AFM or tensiometer force–position data; however, this is inefficient, as the variables Δ*h* and θ_t_ are badly scaled. This issue can be bypassed by scaling the variables to be in the order of one by introducing the following scalings:
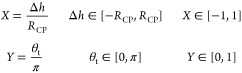


The new set of equations replacing eq 19 and eq 24 is then given by eqs 26a–26c and eqs 27a–27c in terms of the scaled variables *X* and *Y*, which take the role of Δ*h* and θ_t_, respectively, and we note that *X* = cos(α). *X* is the scaled depth of particle immersion and *Y* is the scaled upper phase contact angle.
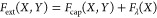
26a
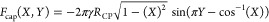
26b
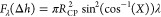
26c
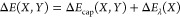
27a
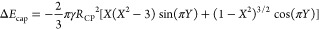
27b
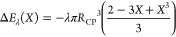
27c

The equation system consisting of eq 26a and eq 27a can be solved efficiently with MATLAB’s *vpasolve* function for any given point along the force–position curve. The height of the three-phase contact line relative to the substrate *h*_TPC_ is then obtained from eq 16 using the location of the colloidal probe center *h*_CP_ that is known from the force–position curve, assuming a negligible deflection of the cantilever and an initially hemispherical shape of the undeformed sessile bubble.

### Calculation of Bubble Interface Profiles

Now it is possible to utilize Sherman’s solution of the Young–Laplace equation to reconstruct the sessile bubbles interface profile during the whole approach–retrace cycle, as all required input parameters are known with the exception of the exact value of λ. The influence of the λ value on the meniscus profile close to the TPCL is small, as it is governed by α, θ_t_ and only the order of magnitude of λ is relevant. During the particle–bubble interaction, a transition between a pinned and a moving TPCL between the bubble and the substrate is possible, which is not explicitly accounted for in eqs 10a–10c. The numerical solution of the parametric eqs 10a–10c for the bubble profile is based on the classic approach of Huh and Scriven,^20^ which solves the boundary value problem of the YL equation as an initial value problem for a given λ, henceforth an appropriate criteria to stop the integration that is satisfying the bubble volume constraint is necessary. The blow up^20^ of the bubble profile is controlled by λ. Due to this, the solver for parametric eqs 10a–10c is nested within an optimization function. If the interface profile of the bubble is known from measurements, λ, α, and θ_t_ can be obtained by fitting eqs 10a–10c using MATLAB’s *lsqcurvefit* function.^19^ However, this is not possible with the data provided by the AFM since the interface profile is not directly available. The deviation of the calculated bubble volume *V*_b_ from the volume of the initially undisturbed bubble  is calculated by  for a given λ. In addition, the end point of the calculated bubble profile has to coincide with the height of the substrate such that distance Δ*y* between the two becomes zero. Therefore, λ is determined by minimizing both the volume error ε_*V*_ and the distance from the substrate Δ*y* by using MATLAB’s *fmincon* function. The final values of α, θ_*t*_ and λ for an experimental data set are obtained by first solving the equations for force eq 26a and work eq 27a with an initial guess of λ_0_ = Δ*P*_L_. Then the equations for the interface profile (eqs 10a–10c) are solved with the obtained parameters within the optimization loop, finding the λ that minimizes both ε_*V*_ and Δ*y*, for the experimental data points. The average parameter λ̅ is calculated and used again to solve the equations for force in eq 26a and work in eq 27a with λ̅ to obtain the final results. The final interface profile is then calculated again with the updated values of α and θ_t_.

## Results and Discussion

### AFM Results

The median force–position curves for the nonequilibrium particle-bubble interactions of a silanized SiO_2_–CP (B1-CP1) or a silanized Al_2_O_3_–CP (B2-CP2 and B3-CP2) with sessile gas bubbles with radii in the range *R*_b_ = 45–80 μm are shown in Figure 2. The force–position curves were obtained from the median force–position curves by assigning the position 0 μm to the point of first contact at which the first force value *F*_ext_ < 0 nN is measured, assuming that the colloidal probe position is then given by *h*_CP,0_ = *R*_b_ + *R*_CP_. The full experimental parameters and results are listed in Table 1. The silanized SiO_2_–CP is smooth and can be seen as perfectly spherical, whereas the silanized Al_2_O_3_–CP also has a spherical shape but possesses the surface irregularities that are common for Al_2_O_3_ manufactured by fused salt electrolysis.

After the film rupture (0) of the liquid film between the sessile bubble and the CP, the particle–bubble aggregate undergoes steps (1)–(6) (Figure 2a). For steps (1) and (2), the piezo drive velocity is *v* < 0 μm/s, and it is reversed at (3) when the predefined maximum piezo push distance has been reached, from whereon *v* > 0 μm/s, as the particle is retracted from the bubble at steps (4)–(6).

(1) Attractive force minimum at the snap-in *F*_ext_ = *F*_snap-in_.
(2) “Equilibrium position” on approach: *F*_ext_ = 0 nN.
(3) Maximum displacement of the CP-particle and reversal of piezo movement: *F*_ext_ = *F*_max_.
(4) “Equilibrium position” on retraction: *F*_ext_ = 0 nN.
(5) Interface profile at the point of the maximum pull-off force: *F*_ext_ = *F*_adh_.
(6) TPCL sliding of the particle after *F*_adh_ was reached, just before the complete detachment: *F*_ext_ < *F*_adh_.
(7) Finally, the complete detachment of the CP from the gas bubble occurs. The cantilever moves back into its undeflected position with *F*_ext_ = 0 nN, and the bubble goes back to its undisturbed equilibrium shape.

All force curves exhibit a snap-in (0) that is characteristic for the attachment of poorly wetted particles to gas-bubbles and results in an attractive force of |*F*_snap-in_|/*R*_CP_ ≥ 50 mN/m (1). The three force–position curves look notably similar; that is, the depth of the snap-in and the maximum pull-off force *F*_adh_ (Figure 2b) do not differ much. The difference between measurements performed with hydrophobic Al_2_O_3_ and hydrophobic SiO_2_ is small, as expected, because their wettability is governed by the silane functionalization. For the interaction with the hydrophobic Al_2_O_3_–CP that possesses a less regular surface, some sudden jumps in the retrace curve are notable (Figure 2b) after the maximum pull-off-force *F*_adh_ has been reached, which indicates alternating stick- and slide-phases of the TPCL on the CP. The measurements with the same CP on the smallest investigated gas bubble with *R*_b_ = 45 μm do not exhibit the same stick-slide behavior (Figure 2c). The smaller size of the bubble in the experiment B3-CP2 shown in Figure 2c leads to an increased nonlinearity of the force–position data that is noticeable around the attractive minima of both the snap-in and the pull-off.

**Figure 3:**
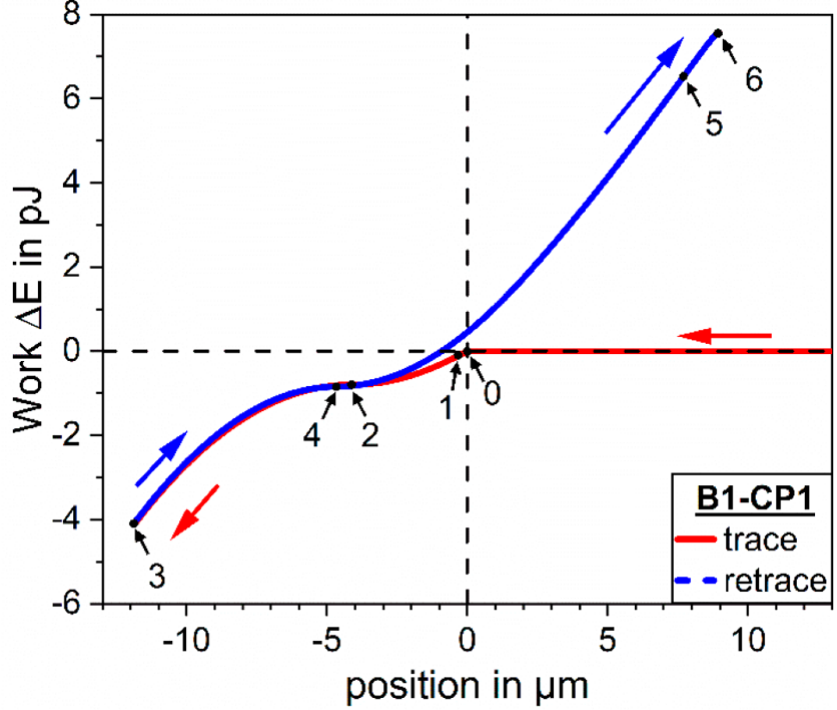
Work Δ*E* required to move the TPCL over the CP-particle was obtained from the force–position data of the experiment B1-CP1 that is shown in Figure 2a.

The work brought up to move the TPCL up and down on the particle was obtained by integration of the experimental force–position curves and is depicted in Figure 3 for experiment B1-CP1 (Figure 2a). It was assumed that the average movement of the TPCL is directed upward during the approach (Δ*E*_*i*_ < 0) and downward during retraction (Δ*E*_*i*_ > 0). With the work-position curves available, all data for the calculation of both the defining angles at the TPCL and the interface profile are given.

### Capillary Force for Flat Interfaces

**Figure 4:**
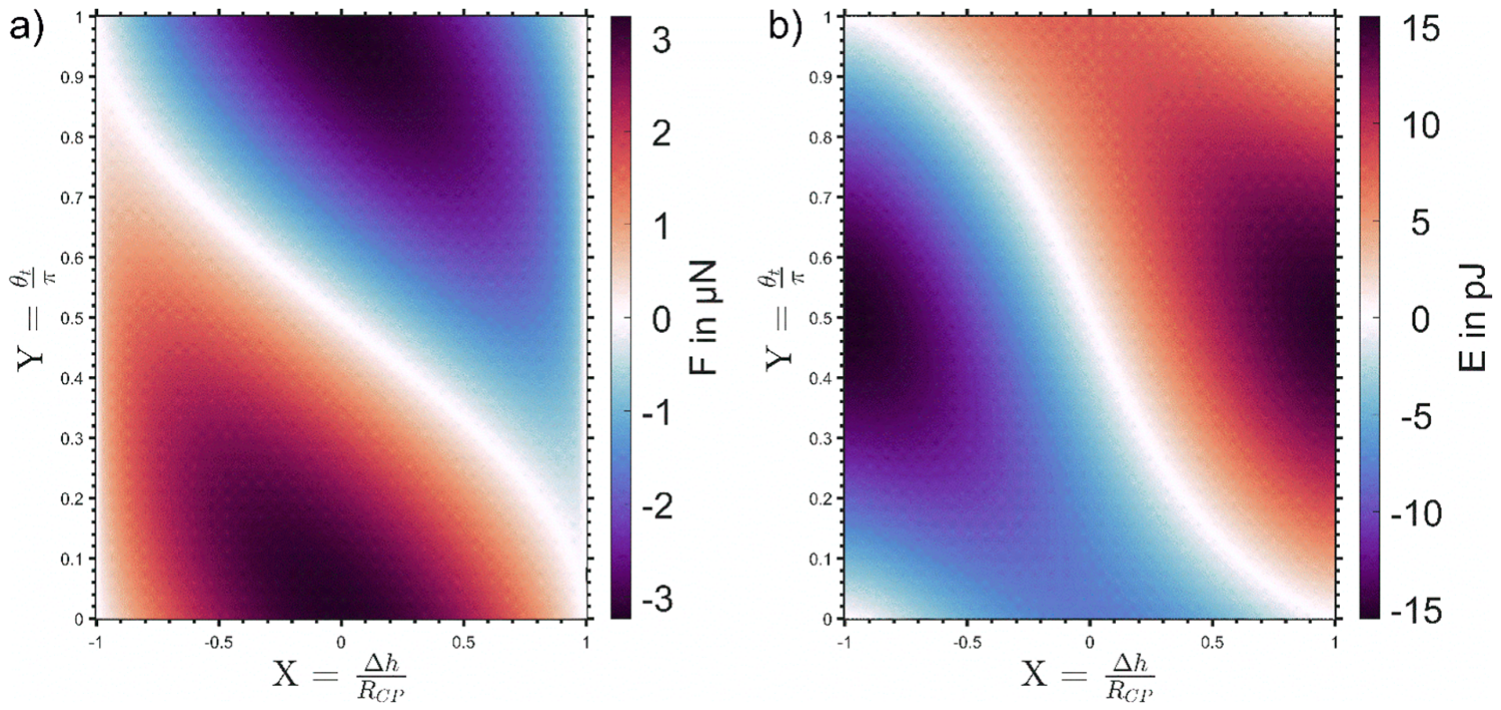
Force- and energy-maps for a particle in contact with an initially flat gas–liquid interface: (a) solution of the vertical component of the capillary force equation (eq 26b) for all combinations of the dimensionless depth of particle immersion *X* and the scaled upper phase contact angle *Y*; (b) energy map indicating the work required to move the TPCL along a particle that is subjected to the vertical component of the capillary force, eq 27b.

The capillary force *F*_cap_ as a solution of eq 26b and the corresponding energy map defined by eq 27b are shown in Figure 4 for all possible combinations of *X* ∈ [−1,1] and *Y* ∈ [0,1]. Both the force map and the energy map are point symmetric around point (*X*,*Y*) = (0,0.5). The distinct attractive minimum (*F* < 0) and repulsive maximum (*F* > 0) in the capillary force map are separated by the zero-force line connecting the bottom right corner (*X*,*Y*) = (1,0) and the upper left corner (*X*,*Y*) = (−1,1) and thereby define the attractive and repulsive combinations of opening angle α and the contact angle θ_*t*_. Intuitively the force becomes zero for |*X*| = 1 when the TPCL reaches either end of the particle.

The energy map in Figure 4b also shows a distinct isoenergy line with *E* = 0 pJ that spans from (*X*,*Y*) = (−1,1) to (*X*,*Y*) = (1,0). From a macroscopic stand point, not yet taking the AFM experiments into account, the particle attachment to the interface can occur from either side of the interface: if the particle is initially completely immersed in the liquid the attachment starts at (*X*,*Y*) = (1,0), and if the particle is completely inside the gas phase it attaches from the opposite side of the interface starting at (*X*,*Y*) = (−1,1).

The energy map specifies the cumulative work that is required to move the TPCL over the particle surface because at the reference point for the integral eq 23a the values of force and work are zero (*F*,*E*) = (0,0). This implies the assumption that the work done to move the TPCL is accumulated in the capillary system that consists of CP and sessile bubble. According to this description the interface cannot relax into its equilibrium shape unless the positive and negative increments of work done to move the TPCL add up to zero. Therefore, the only possible equilibrium position according to the capillary force equation, eq 22, for a flat interface under consideration of eq 23b is reached at (*X*,*Y*) = (0,0.5) or α = θ_t_ = 90°. We note that the zero-force line in Figure 4a corresponds to α = θ_t_ and therefore it covers eq 7 for the contact angle upon attachment to the interface; however, taking into account the work in eq 23b, eq 7 is only fulfilled for α = θ_t_ = 90°. Only forced nonequilibrium (de)-wetting is described by this approach because it only accounts for capillary effects and does not take into account the intrinsic wettability of the particle material as described by the Young equation, eq 1.

### Capillary Force for Particle–Bubble Interactions

**Figure 5:**
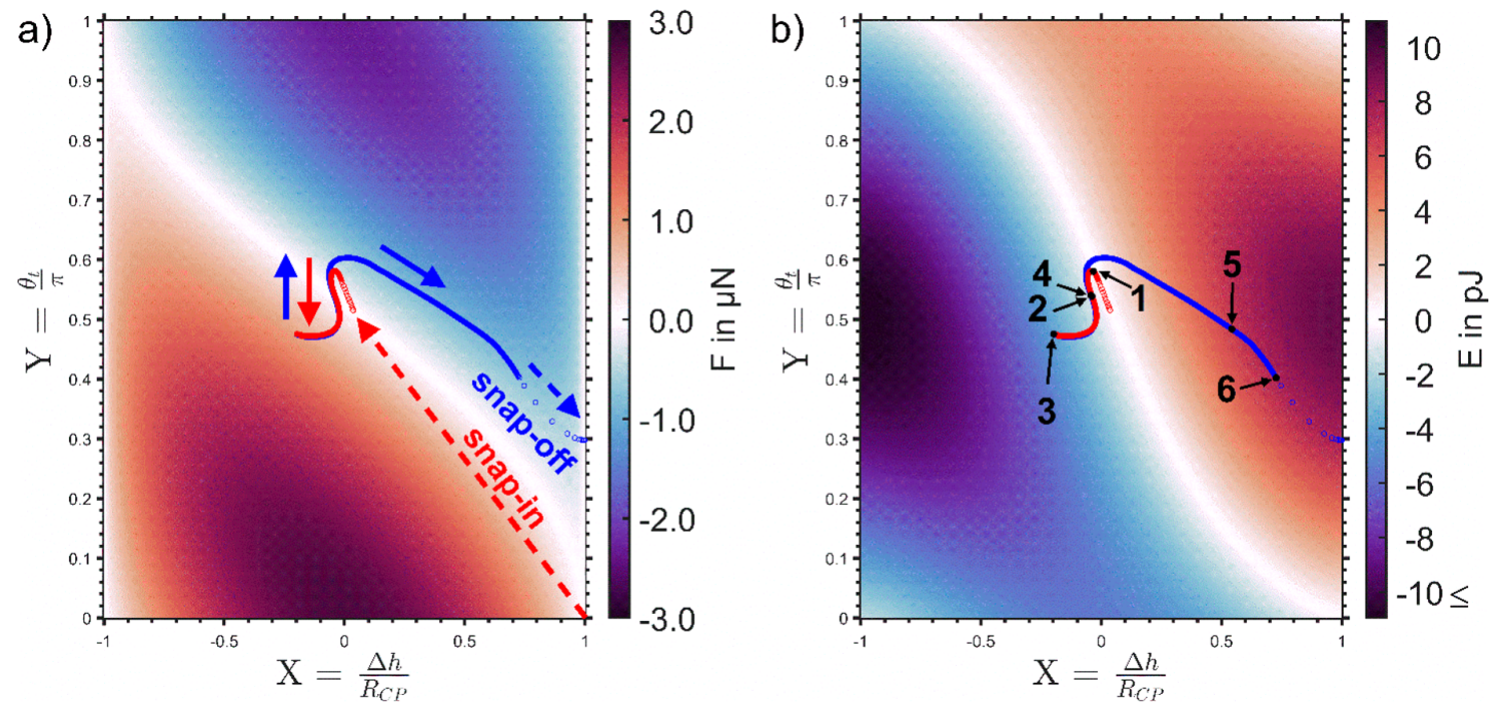
Force and energy maps of a particle in contact with a bubble (*R*_b_ = 68 μm) under consideration of the excess pressure λ that is caused by the interface curvature (λ̅ = 2130.9 Pa). The red (trace) and blue (retrace) markers show the solution of the equation system consisting of eqs 26a and 27a for the interaction of a silanized SiO_2_-CP and a sessile bubble (B1-CP1): (a) solution of the force balance, eq 26a, for all combinations of the dimensionless depth of particle immersion *X* and the scaled upper phase contact angle *Y*; (b) energy map, eq 27a, indicating the work required to move the TPCL over a particle that is subjected to both the vertical component of the capillary force and the pressure force due to the bubble curvature. Description of (1)–(6) is found in the text above.

**Figure 6:**
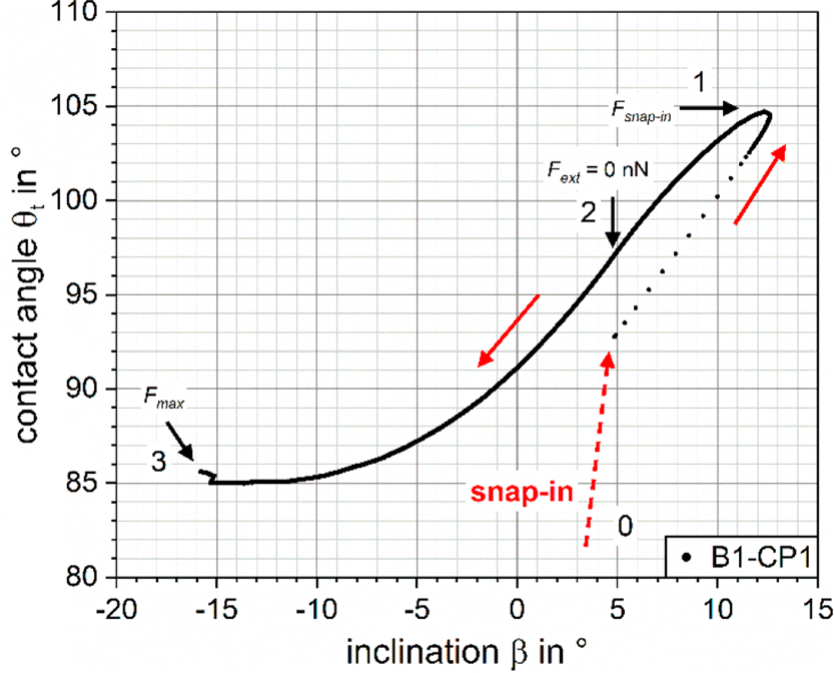
β–θ_t_-curve for the trace of B1-CP1. (0)–(3) correspond to the markers in the force–position curve in Figure 2a and the force-map in Figure 5b.

For small gas bubbles, the additional pressure due to the curvature of the interface can have a significant influence on the particle–bubble interaction and its contribution to the overall force balance, and the work depends on the size of the particle and the depth of penetration into the bubble. As long as *R*_CP_ ≪ *R*_b_, it is not necessary to consider the additional pressure, however, if *R*_CP_ < *R*_b_, that is the particle radius is not much smaller than the bubble, it cannot be neglected, especially if the depth of immersion is expected to be significant. As discussed during the outlining of the model, the increased pressure inside the gas bubble requires additional work to displace the gas Δ*E*_λ_(*V*_cap_) = −λ*V*_cap_ to be done. Therefore, the restoring force of the gas bubble is higher for smaller bubble radii, making it harder for the particle to penetrate into the bubble. Figure 5 shows a pair of force and energy maps similar to the ones depicted in Figure 4 but under consideration of the λ-dependent terms, eq 26c and eq 27c. The sequences of red (trace) and blue (retrace) markers show the solution of the equation system consisting of eqs 26a and 27a for the set of experimental force and work values of experiment B1-CP1. The corresponding θ_t_- and β-values for the trace curve of B1-CP1 are shown in detail in Figure 6. The obtained *X*- and *Y*-values or α- and θ_t_-values for trace and retrace branches are close to each other but do not coincide, which is in line with the observed hysteresis between trace and retrace curves that can be seen at the transition between repulsive and attractive forces for all data depicted in Figure 2. The internal bubble pressure λ̅ = *f*(*R*_b_) varies with the bubble radius *R*_b_; therefore, the resulting force and energy maps (Supporting Information, Figures S3 and S4) for the three experiments are not identical. However, λ̅ values for all three experiments are close to the Laplace pressure (Table 1) and are λ̅ = 1.01 × Δ*P*_L_ for “B1-CP1”, λ̅ = 1.03 × Δ*P*_L_ for “B2-CP2”, and λ̅ = 1.06 × Δ*P*_L_ for the smallest bubble “B3-CP2”.

The first point of the trace curve in Figure 5 is not located at (*X*,*Y*) = (1,0), but rather close to the central intersection of the zero-force and zero-energy curves close to (*X*,*Y*) = (0,0.5). The values of θ_t_ and β for the trace-curve of B1-CP1 are shown in detail in Figure 6. The initial particle dewetting at the snap-in is too fast to be resolved by the AFM measurements with such stiff cantilevers (Table 1) and the TPCL jumps from (*X*,*Y*) = (1,0) (Figure 5) to a point close to the “equilibrium position”. This is reasonable because the dewetting of the particle starts from the initially undeformed bubble interface once the distance between CP and bubble is below a few nanometers, meaning that the bubble deformation upon initial dewetting is driven by the intrinsic particle wettability. For the investigated poorly wetted particles, this results in an upward movement of the TPCL which forces the overall bubble to deform, given the attractive forces are strong enough to lift the TPCL higher onto the CP against the resistance of the surface tension. This is accompanied by an initial increase of the interface inclination β (0 → 1) (Figure 6), reaching its maximum value on approach at the point of the strongest attractive force *F*_snap-in_ (1). At this point, the maximum value of the contact angle θ_t_ = 105° on approach is also reached. After the initial snap-in, the observed contact angle no longer corresponds to the equilibrium contact angle defined by the Young equation, eq 1. Instead, it is now governed by the deformed bubbles effort to take its energetically most favorable shape in the presence of the particle. As the particle is then driven closer to the substrate (1 → 2), β decreases and the first “equilibrium position” is reached at β = 4.8°. Subsequently an increasingly repulsive force is measured until the maximum piezo push distance (3) is reached. At this point, the particle retraction begins and closely follows the *X* and *Y* curves of the trace-curve (Figure 5). When *F* = 0 nN is crossed again (4), the deviation for *X* and *Y* values between trace- and retrace-curves starts to increase and shortly after the maximum value of the contact angle θ_t_ is reached, just after the point where the negative and positive contributions of the work add up to Δ*E* = 0 pJ. From here on, both *X* and *Y*, respectively, α and θ_t_ are decreasing until the particle snaps out of contact (6 → 7).

### TPCL-Movement and Bubble Interface Profile

**Figure 7:**
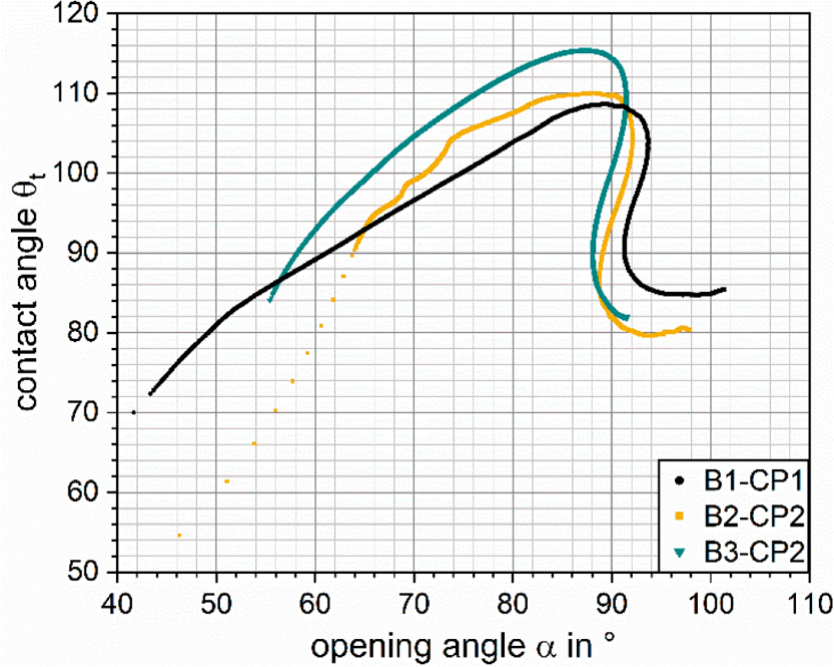
Trajectories through the α–θ_t_-space during particle retraction for the force–position curves shown in Figure 2.

To compare the values of the upper phase contact angle θ_t_ and the opening angle, α, particle immersion, respectively, for the different experiments (Table 1), the results of the retrace part (*v* = 30 μm/s) of the three experiments are shown in Figure 7. The corresponding trace curves are not depicted for the sake of clarity and can be found in the Supporting Information (Figure S5). The maximum values of the upper phase contact angle during retraction for the two bigger bubbles “B1-CP1” (θ_t_ = 109°) and “B2-CP2” (θ_t_ = 110°) do not differ much. For the experiment with the smallest bubble “B3-CP2”, the highest value for the upper phase contact angle is θ_t_ = 115°. Notably, the maximum of θ_t_ is reached at opening angles α close to 90°.

**Figure 8:**
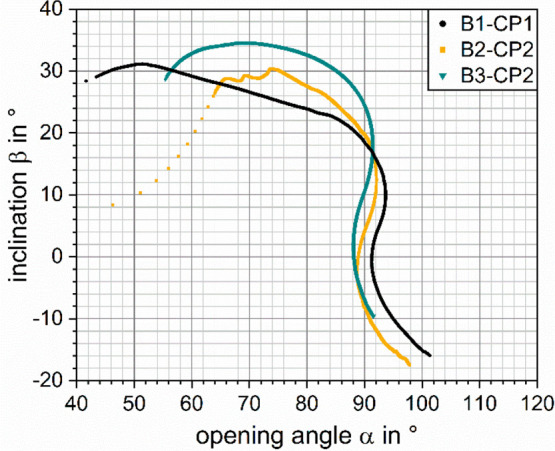
Trajectories through the α–β-space during particle retraction for the force–position curves are shown in Figure 2.

**Figure 9:**
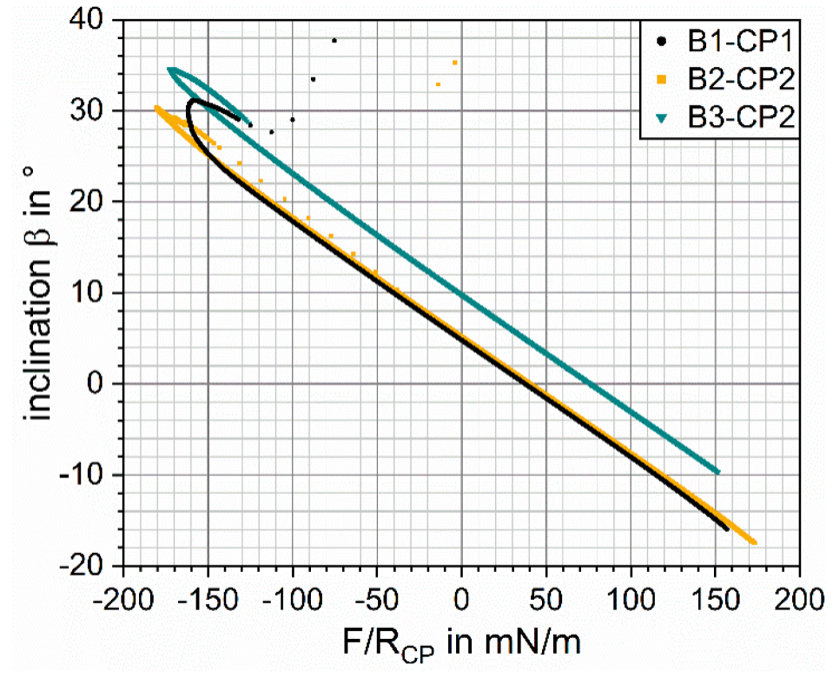
Inclination of the bubble interface β at the TPCL as a function of the measured force scaled by the CP radius, shown for the full approach–retract cycle of the experiments depicted in Figure 2.

The relationship between particle immersion and the local inclination of the bubble interface at the TPCL can be studied by the α–β-plots that are shown in Figure 8 for the retrace part of the three experiments shown in Figure 2 (trace curves, see Supporting Information, Figure S6). The dependency of the interface inclination at the TPCL β on the measured force is shown in Figure 9. Notably, β takes negative values when the CP is pressed into the bubble past its “equilibrium position”, which means that the highest point of the bubble interface is no longer located at (*x*,*y*) = (*r*_TPC_,*h*_TPC_). The smallest β is reached at the maximum piezo-push distance, where the strongest repulsive force of over *F*_max_ > 150 mN/m is measured.

**Figure 10:**
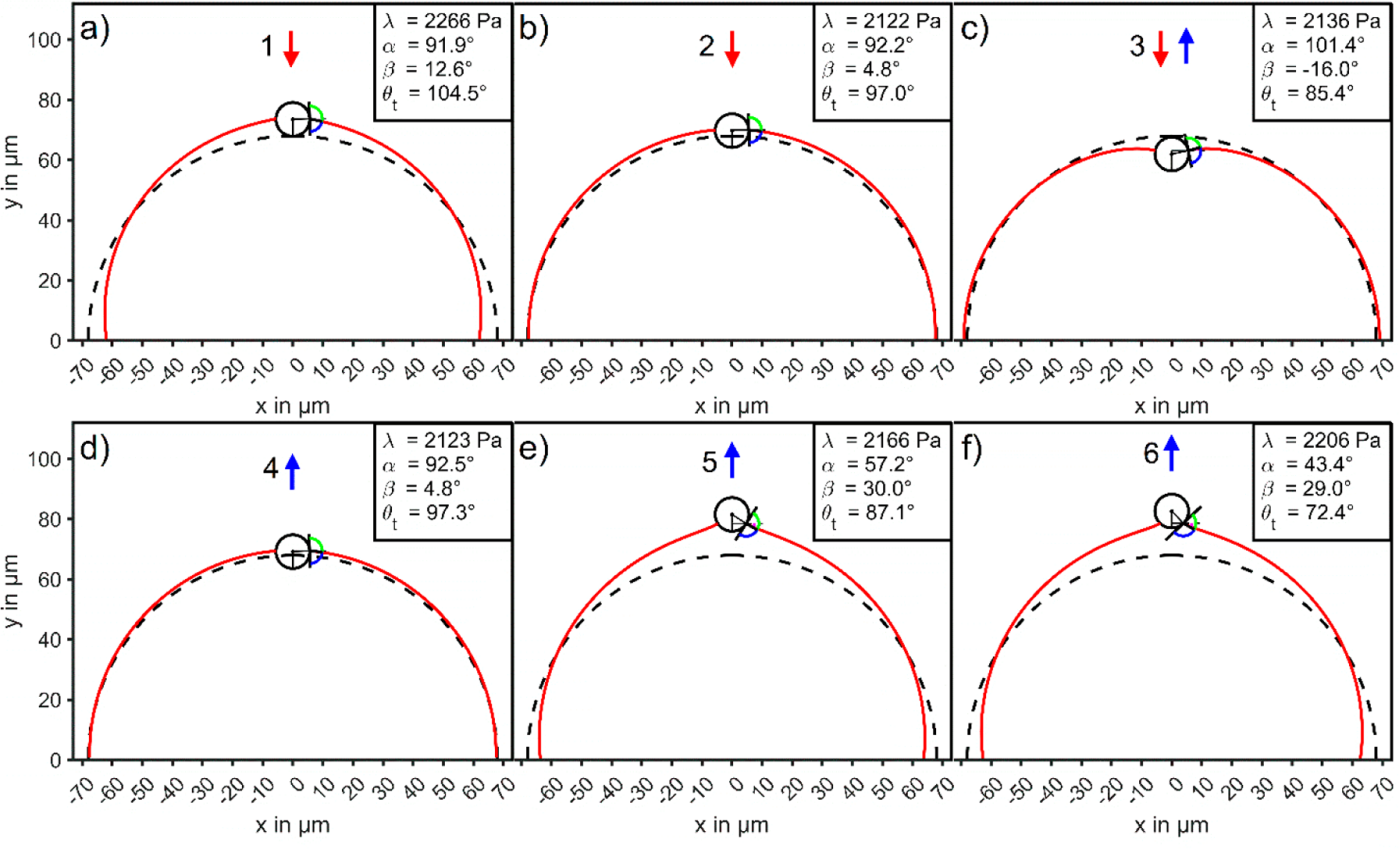
Evolution of the bubble interface profile (red) and TPCL on the CP during the particle–bubble interaction of AFM-experiment B1-CP1. The hemispherical interface profile of the undeformed sessile bubble is given by the dashed line. The contact angle θ_t_ and the opening angle α were obtained by solving eqs 26a and 27a with the force and work information contained in the experimental force–position curves. The interface profile was calculated from the solution of the YL-eq (eqs 10a–10c) for the λ-value that best meets the constant-volume constraint and the contact condition between substrate and bubble at *y* = 0 μm. Numbers 1–6 in parts a-f correspond to the marked points of the experimal force–position curve depicted in Figure 2a and the associated *X*–*Y* trajectories through the force and energy maps shown in Figure 5.

Bubble interface profiles for the points (1)–(6) were calculated from eqs 10a–10c and are shown in Figure 10. Earlier we postulated that the TPCL moves up during the approach (α↑) and down during the retraction (α↓) of the CP. In the region around β = 0°, this assumption is not confirmed as the opening angle α increases by a couple of degrees during retraction and vice versa on approach. At this point the character of the interaction changes as it marks the transition between a concave and a convex shape of the meniscus close to the TPCL. During the whole particle bubble interaction, the TPCL with the substrate can slide in response of the external force acting on the bubble and the need to fulfill the constant volume constraint while undergoing deformation. The likeliness of the substrate-TPCL sliding is higher for small sessile bubbles because the difference between the contact area with the substrate and the contact area with the CP is reduced. This can bring the forces that originate from the CP–bubble–TPCL and substrate–bubble–TPCL in a comparable order of magnitude, which results in an interplay of the two TPCLs (Figure 10). From Figure 9 it can be seen that β is inversely proportional to the scaled interaction force *F*/*R*_CP_ for the complete trace-curve (not visible because of overlap) and the majority of the retrace-curve. This linear β-(*F*/*R*_CP_) dependency is observed for all three experiments; it is independent of the bubble radius in the investigated range *R*_b_ = 45–80 μm and well described by  for *F* ≥ 0.9 × *F*_adh_. Note that the two “equilibrium positions” on approach (2) and retraction (4) are characterized by the same inclination of the interface at the TPCL β.

At the highest measured attractive force *F*_adh_, the inclination of the interface at the TPCL reaches its highest value β_max_. After this maximum pull-off force, *F*_adh_ is reached, β starts to decrease (Figure 9) because from this point on it is energetically favorable for the bubble to go back to its unperturbed equilibrium shape instead of stretching further to remain in contact with the CP, therefore marking the tipping point of the stable particle–bubble contact. This is accompanied by a decreasing opening angle α and upper phase contact angle θ_t_ until the sudden snap-off occurs, at which the measured force becomes zero and the contact between CP and bubble is lost. The amount of necking of the meniscus during particle retraction is governed by the maximum in the energy map Figure 5b that is located at *X* = 1, as there are multiple combinations of *X* and *Y* that can fulfill eq 26a alone. The area under the force–position curve is the cumulative work done on the capillary system and decides at which *Y*-value the CP snaps out of contact. A higher value of Δ*E* at the end of the interaction results in a greater contact angle θ_t_ and therefore makes necking of the meniscus more likely.

## Conclusion

A method for the calculation of contact-angles, the three-phase contact line position, and bubble-interface profiles from CP-AFM data was proposed. The calculation requires a set of force-, work-, and CP-position data obtained from force–position curves. The calculation of the bubble interface-profile from the Young–Laplace-Equation is optional because the excess pressure λ that determines the bubble shape is sufficiently well approximated from the Laplace pressure Δ*P*_L_ because λ̅ does not exceed Δ*P*_L_ by more than 6% for bubbles with radii *R*_b_ = 45–80 μm. However, the bubble–interface profiles are required to completely understand the particle-bubble interaction because sessile bubbles with small radii undergo strong deformation, and the TPCL-position on the CP is a result of the interplay between the TPCL on the particle and the TPCL with the substrate.

The applicability of the method was demonstrated in CP-AFM experiments between sessile gas bubbles and hydrophobic SiO_2_ or hydrophobic Al_2_O_3_ particles. The influence of the bubble radius in the range of *R*_b_ = 45–80 μm on the interaction is small. Differences in the morphology of the CP-particles have little effect on the interaction because their influence on the work is insignificant.

The evaluation of the TPCL-movement and the contact angle between particle and bubble requires consideration of both the force and the work done on the capillary system. Once the particle is attached to the gas bubble the interaction is governed by the inclination of the bubble interface profile at the three-phase contact line β, which is directly proportional to the negative value of the force measured by AFM – *F*_ext_/*R*_CP_, until 90% of the pull of force value *F*_adh_ is reached. It can be concluded that the nonequilibrium interaction is governed by the deformation of the bubble; thus, the surface tension γ is the crucial parameter determining the stability of particle–bubble aggregates. The proposed method should be applied to systems with chemical composition relevant for flotation, as it holds the potential to highlight differences in particle-bubble aggregate stability.

## Supporting Information Available

Additional plots of force and energy maps and detailed plots of the three-phase contact line characteristics on trace and retrace (PDF)
Definitions (PDF)

## Abbreviations

AFM: atomic force microscopy
CP: colloidal probe
FD: force–distance
TPCL: three-phase contact line
YL: Young–Laplace
