# Supplementary material for: Particle–Bubble Interactions: an Investigation of the Three-Phase Contact Line by Atomic Force Microscopy
Source: Langmuir. 2023 Sep 14;39(38):13630–40. doi: 10.1021/acs.langmuir.3c01781 (PMC10537429; doi:10.1021/acs.langmuir.3c01781)
Supplement: Supplementary file 1 — la3c01781_si_001.pdf [file la3c01781_si_001.pdf]

Particle-Bubble Interactions: an Investigation of the Three-Phase Contact Line by Atomic Force Microscopy

Supporting Information

Jan Nicklas, Lisa Ditscherlein, Urs A. Peuker*

TU Bergakademie Freiberg, Institute of Mechanical Process Engineering and Mineral Processing; Agricolastraße 1, 09599 Freiberg, Germany

CORRESPONDING AUTHORS: urs.peuker@mvtat.tu-freiberg.de

jan.nicklas@mvtat.tu-freiberg.de

<u>Content</u>

1. Overview over CP-AFM experiments **S2**

2. Force- and energy maps **S3**

3. Movement of the three-phase contact line **S5**

1. Overview over CP-AFM Experiments

This section includes an annotated version of Figure 2, with the numbers highlighted for orientation in the force- and energy maps presented in the Results and Discussion Section.

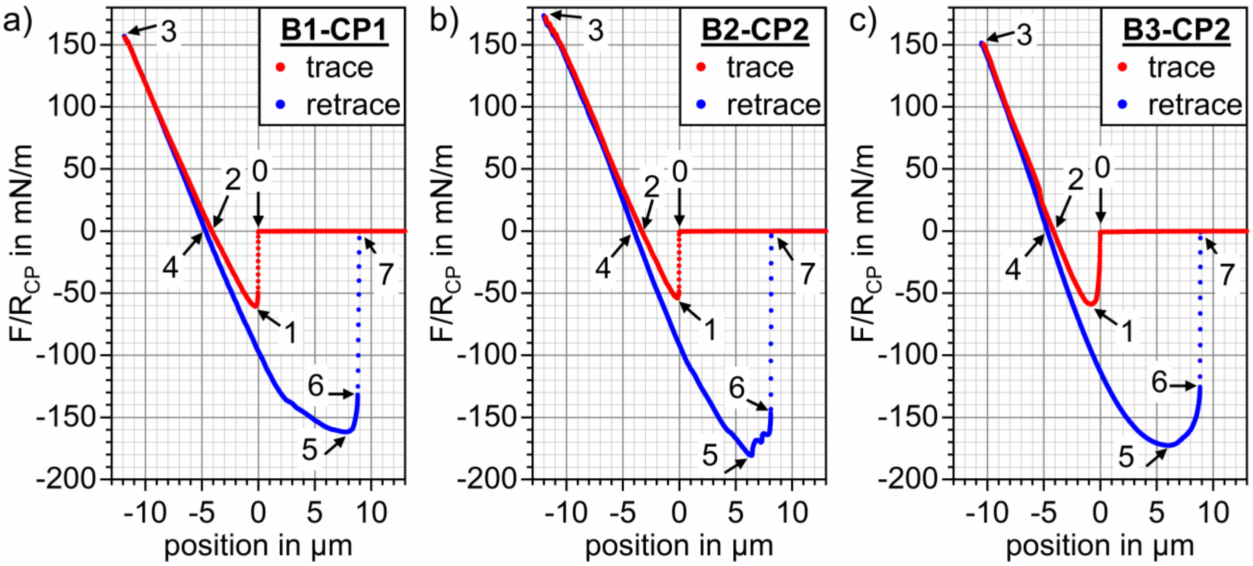

Figure S1. Force-position curves for the interaction of hydrophobic particles and sessile gas bubbles at a piezo drive velocity of v = ± 30 µm/s. For experimental paramters see Table 1.

2. Force- and Energy Maps

For small gas bubbles the Laplace pressure and therefore the parameter for the excess pressure $\bar{\lambda}$ increases. The resulting energy maps according to Eq.(27a) for bubbles of different size are not identical. An increase of $\bar{\lambda}$ shifts the iso-energy line with $E = 0$ pJ that connects the points $(X, Y) = (-1,1)$ and $(X, Y) = (1,0)$ to higher values of the scaled depth of immersion X. The combined solution of the equations for force Eq. (26a) and energy Eq.(27a) for a given set of experimental data, therefore is also affected.

The combined force- and energy-maps for all three experiments are shown in Figure S2 (B1-CP1), Figure S3 (B2-CP2) and Figure S4 (B3-CP2). The numbers (1-6) correspond to the point on the force-position curves in Figure S1a-c.

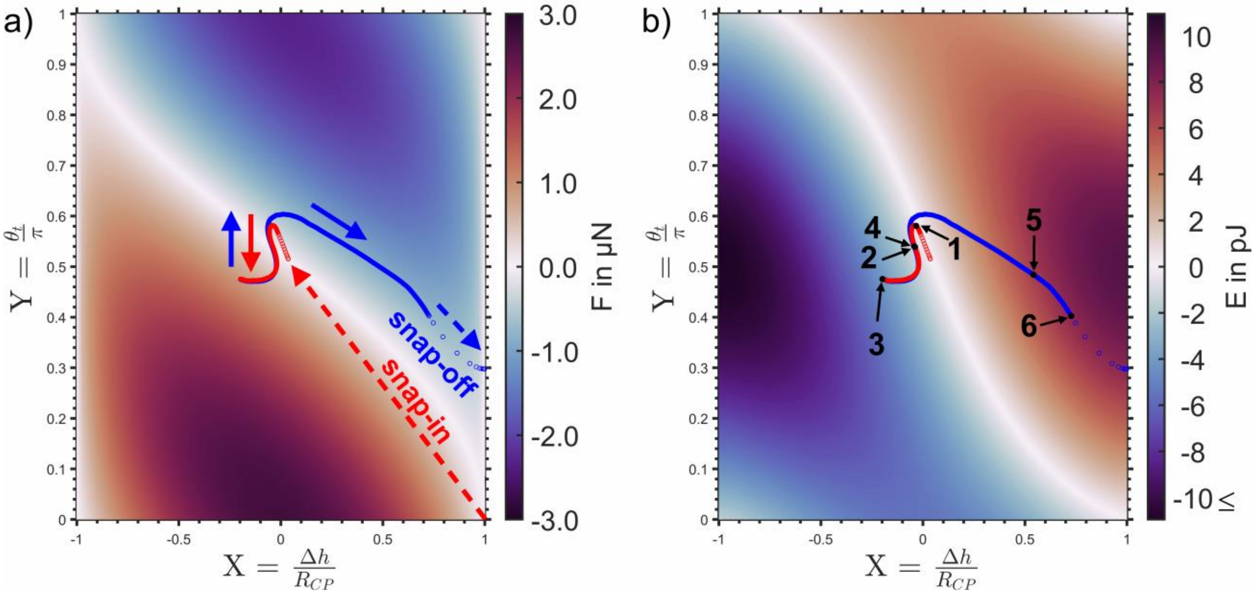

Figure S2. a) Force- and b) energy-maps calculated for the experiment B1-CP1 (Figure. S1a) with $\bar{\lambda}$ = 2130.9 Pa. The markers indicate the values of the scaled depth of immersion X and the scaled upper phase contact angle Y during trace (red) and retrace (blue). (1-6) correspond to (1-6) in Figure S1a.

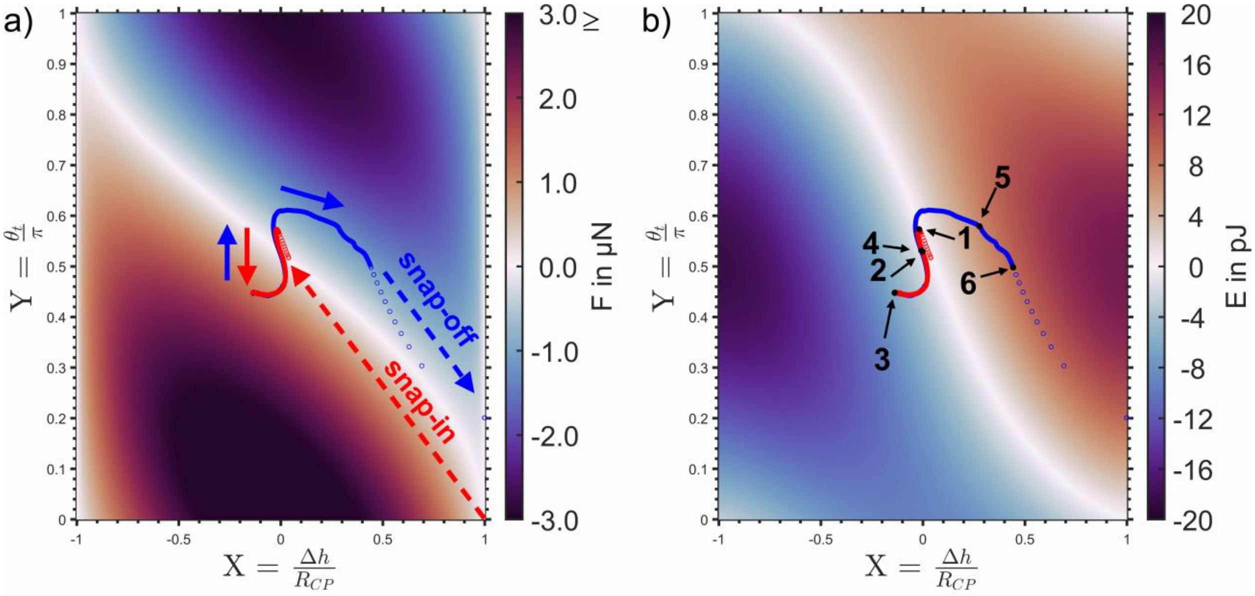

Figure S3. a) Force- and b) energy-maps calculated for the experiment B2-CP2 (Figure. S1b) with $\bar{\lambda}$ = 1845.0 Pa. The markers indicate the values of the scaled depth of immersion X and the scaled upper phase contact angle Y during trace (red) and retrace (blue). (1-6) correspond to (1-6) in Figure S1b.

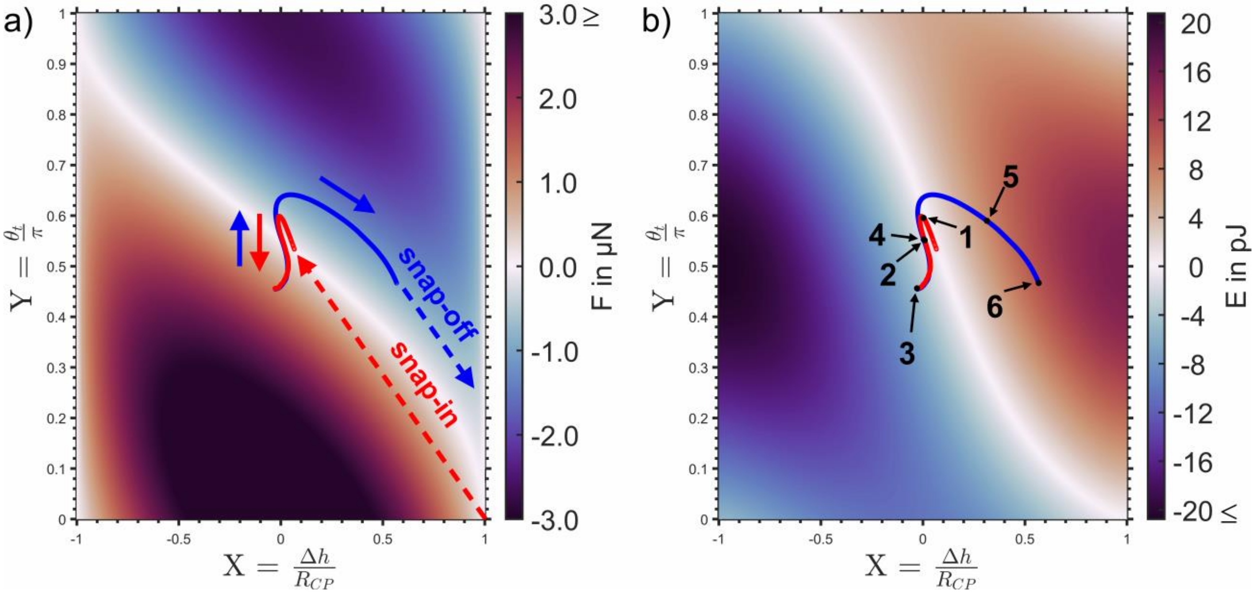

Figure S4. a) Force- and b) energy-maps calculated for the experiment B3-CP2 (Figure. S1c) with $\bar{\lambda}$ = 3355.2 Pa. The markers indicate the values of the scaled depth of immersion X and the scaled upper phase contact angle Y during trace (red) and retrace (blue). (1-6) correspond to (1-6) in Figure S1c.

3. Movement of the Three-Phase Contact Line

In Figure S5 the $\boldsymbol{\alpha}$-$\boldsymbol{\theta_t}$-plots for the three experiments (Figure S1) are shown, similar to the combined plots of the retrace parts in Figure 7, however this time the trace curves (red) are shown in addition to the retrace portion (blue). The trace and retrace curves are almost identical and overlapping between the two so called ‘equilibrium positions’ (2) and (4) for all experiments. No sudden changes in neither contact angle, or opening angle are observed when the direction of the CP-movement is reversed at the point of maximum piezo-push-distance (3). For completeness the corresponding α-β-plots including trace and retrace data are shown in Figure S6.

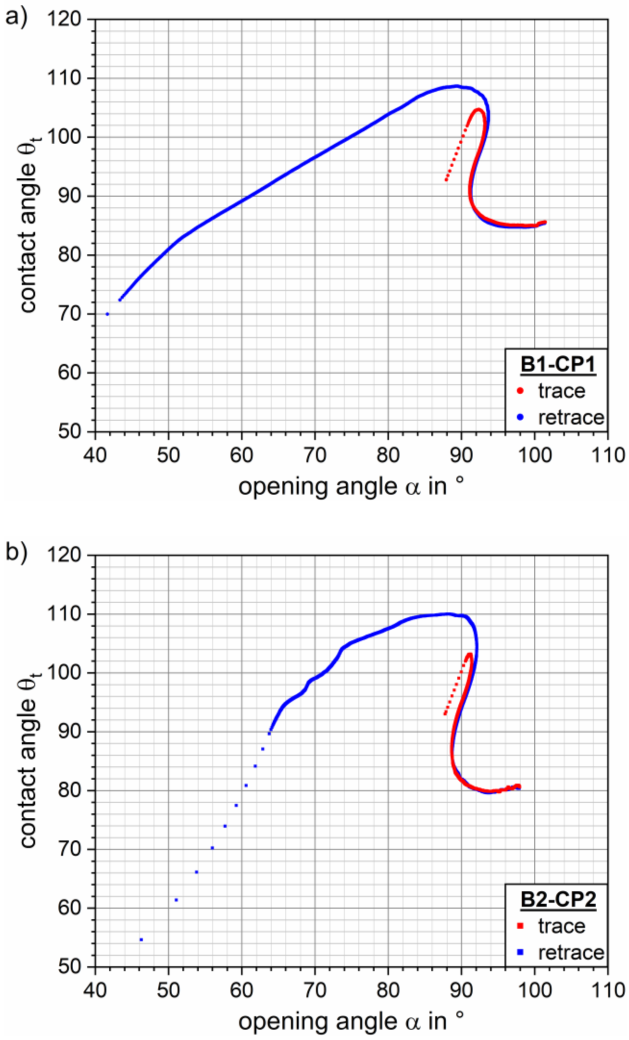

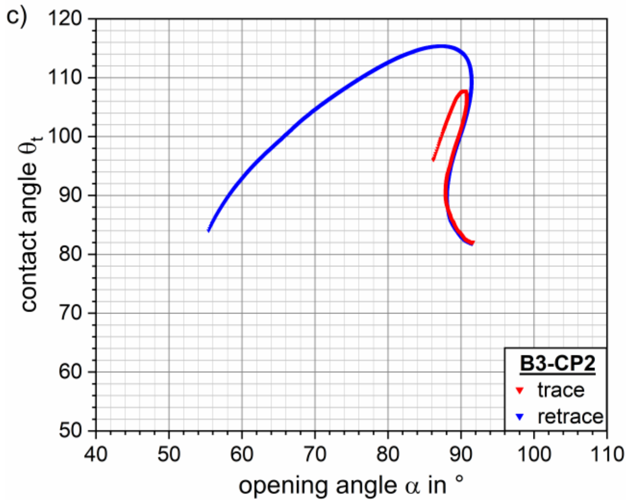

Figure S5. Trajectories through the α-θ_t-space during trace (red) and retrace (blue) for the force-position curves shown in Figure S1: a) B1-CP1, b) B2-CP2, c) B3-CP2

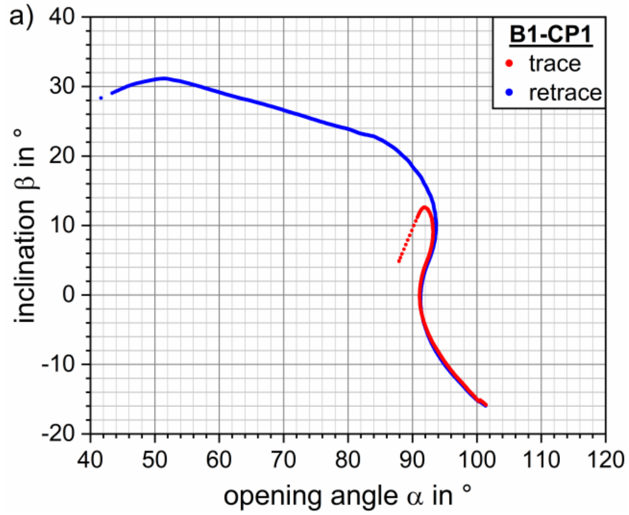

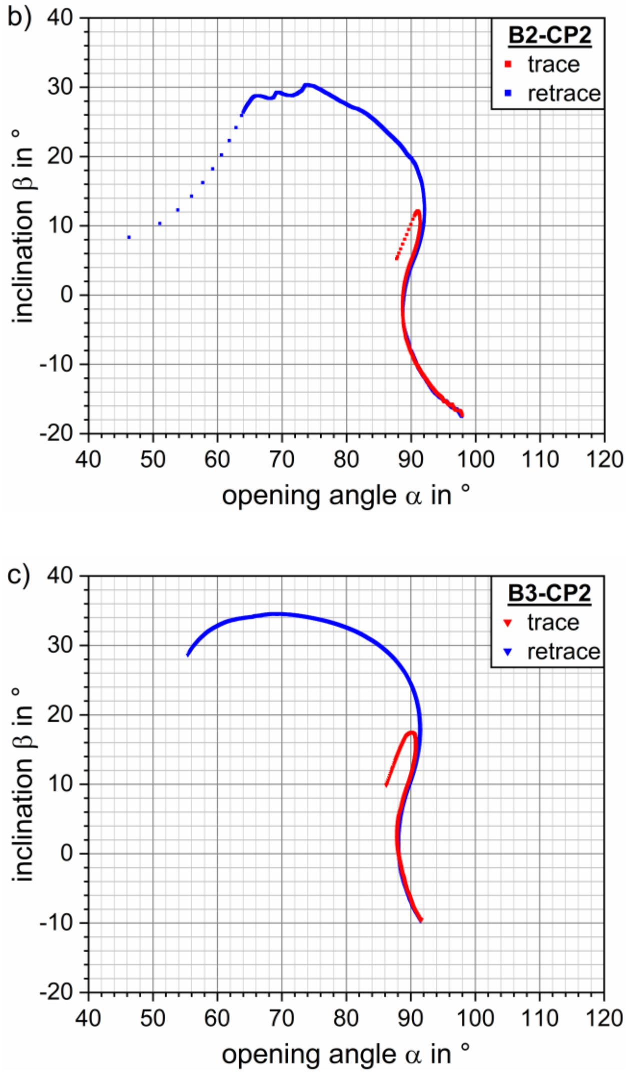

Figure S6. Trajectories through the α-β-space during trace (red) and retrace (blue) for the force-position curves shown in Figure S1: a) B1-CP1, b) B2-CP2, c) B3-CP2
