# Supplementary material for: Particle–Bubble Interactions: an Investigation of the Three-Phase Contact Line by Atomic Force Microscopy
Source: Langmuir. 2023 Sep 14;39(38):13630–40. doi: 10.1021/acs.langmuir.3c01781 (PMC10537429; doi:10.1021/acs.langmuir.3c01781)
Supplement: Supplementary file 2 — la3c01781_si_002.pdf [file la3c01781_si_002.pdf]

SYMBOL/ABBR.	UNIT	MEANING
AFM		atomic force microscope
CP		colloidal probe
FD		force-distance
SRYL		Stokes-Reynolds-Young-Laplace
TPCL		three-phase-contact-line
YL		Young-Laplace-Equation
α	rad / °	opening angle
β	rad / °	interface inclination
ΔE	J	work
ΔE_{λ}	J	pressure-volume-work
ΔE_{cap}	J	work due to surface-tension
ε_V	-	volume error
Δh	m	position of the three-phase-contact-line relative to the center of the colloidal-probe-particle
ΔP_L	Pa	Laplace pressure
Δy	m	distance to the substrate
$\Delta z_{snap-in}$	m	snap-in distance
λ	Pa	excess pressure / Lagrange multiplier
ϕ	rad / °	slope
θ	rad / °	contact angle
θ_{at}	rad / °	contact angle at attachment
θ_b	rad / °	bottom phase contact angle
θ_t	rad / °	top phase contact angle
γ	N/m	interface-tension
γ_{sl}	N/m	surface-energy solid-liquid
γ_{sg}	N/m	surface-energy solid-gas
γ_{gl}	N/m	surface-tension solid-liquid
E_{adh}	J	work of adhesion
F_{λ}	N	force due to excess pressure
F_{adh}	N	force of adhesion / maximum pull-off-force
F_{cap}	N	vertical component of the capillary force
F_b	N	buoyancy force
F_{ext}	N	force acting on the colloidal-probe-particle
F_g	N	gravitational force

F_{max}	N	maximum repulsive force
F_p	N	pressure force
$F_{snap-in}$	N	force at the snap-in
g	m/s^2	gravitational constant
h_{CP}	m	position of the colloidal-probe-center relative to the the substrate
h_{TPC}	m	height of the three-phase-contact-line relative to the substrate
$h(r)$	m	interface hight
k_c	N/m	cantilever spring constant
l_{cap}	m	capillary length
r	m	radius
r_{TPC}	m	radius of the three-phase-contact-line
R_b	m	bubble radius
R_{CP}	m	radius of the colloidal-probe particle
s		parameter
v	m/s	piezo drive velocity
V_b	m^3	bubble volume
$V_{b,c0}$	m^3	bubble volume of the undeformed bubble
V_{cap}	m^3	volume of the de-wetted particle cap
x	m	position
x^*	-	dimensionless position
X	-	scaled depth of particle immersion
y	-	position
y^*	-	dimensionless position
Y	-	scaled upper phase contact angle
